# The Value of Cranial CT Imaging in Patients With Headache at the Emergency Department

**DOI:** 10.3389/fneur.2021.663353

**Published:** 2021-05-10

**Authors:** Cynthia M. C. Lemmens, M. Christien van der Linden, Korné Jellema

**Affiliations:** ^1^Department of Neurology, Haaglanden Medical Center, The Hague, Netherlands; ^2^Emergency Department, Haaglanden Medical Center, The Hague, Netherlands

**Keywords:** headache, emergency department, cranial imaging, computed tomography, migraine, acute headache

## Abstract

**Background:** Headache is among the most prevalent complaints in patients presenting to the emergency department (ED). Clinicians are faced with the difficult task to differentiate primary (benign) from secondary headache disorders, since no international guidelines currently exist of clinical indicators for neuroimaging in headache patients.

**Methods:** We performed a retrospective review of 501 patients who presented at the ED with headache as a primary complaint between April 2018 and December 2018. Primary outcomes included the amount of diagnostic imaging, the different conclusions provided by diagnostic imaging, and the clinical factors associated with abnormal imaging results.

**Results:** About half of the patients were diagnosed with a primary headache disorder. Cranial CT imaging at the ED was performed regularly (61% of the patients) and led to the diagnosis of underlying pathology in 1 in 7.6 patients. In a multivariate model, factors significantly associated with abnormal cranial CT results were age 50 years or older, presentation within 1 h after headache onset, clinical history of aphasia, and focal neurological deficit at examination.

**Conclusions:** As separate clinical characteristics have limited value in detecting severe underlying headache disorders, cranial imaging is regularly performed in the ED. Clinical prediction model tools applied to headache patients may identify patients at risk of intracranial pathology prior to diagnostic imaging and reduce cranial imaging in the future.

## Background

Headache is among the most common complaints in patients evaluated in the emergency department (ED), accounting for ~2.3% of all ED visits (1–3). Clinicians may experience difficulty in differentiating primary (benign) headache disorders from secondary causes requiring prompt neuroimaging in the emergency setting (4). The majority of patients admitted to the ED with headache are diagnosed with a benign primary headache disorder. Nevertheless, about 10% of headache patients are diagnosed with severe secondary headache disorders including trauma, hemorrhage, vascular pathology, infection, or malignancy (5–7). Clinical clues for secondary headache causes can be obtained from vital signs and extensive neurological examination; however, these may be absent in a substantial amount of patients with intracranial pathology. Misdiagnosis of secondary headache disorders has severe consequences, as harmful underlying pathology may be inadequately treated, leading to disability or even mortality. As a result, additional imaging including computed tomography (CT) and magnetic resonance imaging (MRI) is frequently performed to rule out severe secondary headache causes. These tests pose a burden to both patients (e.g., radiation, risk of complications/contrast allergies, and discomfort) and healthcare providers (e.g., increased length of stay at the ED and overcrowding at the ED) and leads to considerable healthcare costs. Moreover, incidental findings on cranial imaging unrelated to headache may cause unnecessary anxiety in these patients. ED clinicians must therefore weigh the costs of additional analyses against the risk of misdiagnosing underlying pathology. There are currently no (inter)national guidelines that describe the clinical criteria for performing cranial CT imaging in non-traumatic headache patients, although “red flags” have been identified in the SNNOOP10 list (systemic symptoms, neurologic symptoms or signs, sudden onset or onset after the age of 40 years, and change of headache pattern) (8). Clinical characteristics in headache patients that indicate specialized neurological evaluation (according to the Dutch general practitioner guidelines) are a new type of headache in patients 50 years or older, acute onset of headache, neck stiffness, fever, headache combined with morning vomiting, immunocompromised patients or history of cancer, and progressive headache within 6 weeks following head trauma (9). The primary aim of this study was to assess the yield of CT imaging in patients presenting with headache at the ED while applying current local guidelines and red flags. As a second aim, we determined the prognostic value of clinical variables in relation to abnormalities on CT imaging, which could ultimately contribute to clinical decision rules of neuroimaging in patients presenting with headache at the ED.

## Methods

In this retrospective cohort study, we included consecutive patients with headache as primary registered complaint (i.e., either referred by the general practitioner or reported by the patient) who were evaluated by the neurologist or emergency physician at the ED in the Haaglanden Medical Centre in The Hague, a large teaching hospital in The Netherlands with 55,000 annual ED visits, from April 2018 to December 2018. Patients were identified through a manual search of the electronic patient records of all ED visits during this period. Patients with headache secondary to recent head injury (within 2 days) were excluded. In case of multiple ED visits for headache complaints by the same patient, the ED presentation at which the final diagnosis was determined was included for analysis. Primary evaluation of the patient was performed by either an ED consultant/resident or a neurology resident. The final diagnosis and further policy regarding treatment or follow-up was established by a supervising neurologist. Patient characteristics indicative for neuroimaging (red flags) in the local protocol included neurological deficits at examination, acute and severe headache, fever, progressive headache, pregnancy or puerperium, history of oncological disease, coagulopathy, and immunosuppression.

We extracted data from electronic records on patient characteristics including age, sex, medical history, neurological examination, additional diagnostic tests (cranial CT, MRI, laboratory results, and lumbar puncture), and diagnosis at ED discharge. Focal neurological deficits were recorded as 1. objectifiable symptoms, such as hemiparesis or facial weakness, 2. solely (unilateral) sensory deficit, and 3. symptoms indicative of conversion disorder (e.g., monocular diplopia, dysphonia, dissociative collapse or non-epileptic seizure, or functional limb paresis with discrepancies during motor testing).

We collected data on cranial imaging reports as diagnosed by a (neuro-)radiologist at the time of presentation at the ED. CT results were considered abnormal if one of the following intracranial lesions was present: intracerebral hemorrhage or recent ischemic stroke, subarachnoid hemorrhage, subdural or epidural hematoma, signs of intracranial hypertension, hydrocephalus, abscess, or new neoplasms. We extracted results of computed tomography venography (CTV) and angiography (CTA) if applicable. Data on MRI scans performed within 1 month after ED visit and lumbar puncture results were collected. The final diagnosis was derived from ED discharge letters or follow-up (outpatient clinic) visits if available.

We performed statistical testing using the Chi-square test and unadjusted odds ratios (ORs) for dichotomous data to assess univariate associations between clinical characteristics and abnormal CT findings. Statistical significance was set at a *p* ≤ 0.05. All variables that were associated during univariate analyses with pathological findings at brain CT with *p* ≤ 0.20 were entered into a multivariate logistic regression model to determine adjusted effect sizes to predict abnormal CT findings. We calculated adjusted ORs and their 95% confidence intervals (CIs). We performed all statistical analyses with SPSS Statistics for Windows version 26 (IBM SPSS Statistics for Windows, version 22.0; IBM Corp. Armonk, NY, USA).

The study protocol was in accordance with the current STROBE guidelines, and a formal ethical evaluation was waived due to the retrospective design of our study by the accredited Medical Research Ethics Committee (MREC) Leiden/The Hague/Delft. All research data were anonymized and accessible only by the primary researcher.

## Results

We evaluated 501 patients with headache as primary complaint presenting to the ED. Median age was 42 years (aged 13–91 years); 325 patients were female (65%). On average, two patients per day were assessed for headache complaints by the neurologist during the 10-month period. In 6% of the cases, the patient was reassessed at the ED for persisting headache within 1 week. Twenty percent of the patients visited the ED multiple times for complaints of headache within the last 5 years. Sixty percent of the patients reported a history of headache. Acute onset of headache (maximum headache intensity within 5 min) was reported in 143 patients (29%). Symptoms of clinical history and neurological examination are presented in Table 1. Newly originated focal neurologic deficits were observed in 107 patients (21%), including patients suspect of functional neurological deficit and isolated sensory deficit.

**Table 1:** Clinical characteristics of patients and correlation to intracranial pathology on cranial CT.

	**Characteristic (present/recorded in dossier)**	**Abnormal CT results in relation to clinical sign; OR (95% CI)**
**Clinical history**		
Duration <1 h	42/501	10/34; OR 3.3 (1.5–7.6)
Acute onset	143/501	16/117; OR 1.1 (0.55–2.1)
Episodic headache	68/501	1/33; OR 0.2 (0.03–1.4)
Photophobia	113/212	5/71; OR 0.8 (0.22–2.9)
Phonophobia	57/147	2/40; OR 0.5 (0.09–2.6)
Visual deficits/aura	131/413	8/88; OR 0.6 (0.25–1.4)
Nausea	284/453	30/138; OR 2.1 (0.9–4.8)
Fever	45/501	2/20; OR 0.7 (0.16–3.2)
(Transient) aphasia	26/501	10/22; OR 7.0 (2.8–17.6)
(Transient) paresis	30/501	7/23; OR 3.3 (1.3–8.6)
(Transient) sensory deficit	62/501	5/40; OR 0.9 (0.34–2.5)
**Neurological examination**		
Focal neurological deficit	107/501	17/82; OR 2.3 (1.1–4.5)
Objectifiable deficit	52/107	16/40; OR 5.4 (2.6–11)
Suspect of conversion disorder	20/107	
Isolated sensory deficit	35/107	
Hypertension^A^	147/501	17/111; OR 1.3 (0.7–2.6)
Neck stiffness	14/262	
Papilledema at fundoscopy	3/45	

*CT, cranial imaging; OR, odds ratio; CI, confidence interval*.

A *Defined as systolic blood pressure >160 mmHg and/or diastolic blood pressure >110 mmHg*.

An overview of all diagnoses is presented in Table 2. A primary headache disorder was -diagnosed in 240 patients (48%). The most frequent diagnosis was tension type headache in 97 patients (19%). In 59 patients (12%), a severe secondary cause (vascular disorder, bacterial meningitis, or cerebral neoplasm) was diagnosed.

**Table 2:** Diagnoses/headache etiologies.

	**Patients (*N*)**	**Percentage (%)**
**Primary headache syndromes**	**240**	**48**
Tension headache	97	19
Migraine	81	16
Thunderclap headache	44	8.7
Cluster headache/hemicrania continua	13	2.6
Trigeminal neuralgia	3	0.6
Postcoital headache	2	0.4
**Secondary headache syndromes**	**261**	**52**
Central nervous system infection		
Viral meningitis/encephalitis	26	5.2
Bacterial meningitis	3	0.6
Neurosyphilis/basal meningitis	2	0.4
Cerebrovascular disease		
Subarachnoid hemorrhage	17	3.4
Ischemic stroke/transient ischemic attack	12	2.4
Intracerebral hemorrhage	7	1.4
Cerebral venous sinus thrombosis	5	1.0
Carotid/vertebral artery dissection	3	0.6
Reversible cerebral vasoconstriction syndrome	3	0.6
Neoplasm of the brain		
Intracerebral malign mass/leptomeningeal	9	1.8
carcinomatosis		
Systemic/local infectious or inflammatory disorder		
Parainfectious headache	41	8.2
Sinusitis	14	2.8
Headache secondary to facial	5	1.0
inflammation/infection		
Temporal arteritis	3	0.6
Other etiologies		
Postcommotional headache (trauma >2 days	25	5.0
prior to presentation)		
Conversion syndrome/hyperventilation	15	3.0
Medication induced headache	14	2.8
Peripheral vestibular syndrome	13	2.6
Post-lumbar puncture headache/intracranial	10	2.0
hypotension syndrome		
Hypertensive crisis	10	2.0
Ocular etiology	6	1.2
Idiopathic intracranial hypertension	5	1.0
Metabolic dysregulation (anemia/dehydration)	5	1.0
Other: alcohol/drug-induced headache,	8	1.6
epileptic seizure, hydrocephalus, vasculitis,		
pituitary gland apoplexy, and Bell's palsy		

Cranial imaging CT was performed in 305 patients (61%); results were deemed by the radiologist as normal in 265 patients (87%). In 40 patients (13%), intracranial lesions were detected, including intracerebral hemorrhage or ischemia, subarachnoid hemorrhage, subdural hematoma, signs of intracranial hypertension, new space-occupying lesions, hydrocephalus, or previously diagnosed tumors with intralesional bleeding or progression of perilesional edema. In four of these 40 patients, the observed intracerebral lesion was not considered to be a sufficient cause of the headache, including three patients with perilesional edema or newly observed neoplasm and one patient with ischemic stroke.

**Figure 1:**
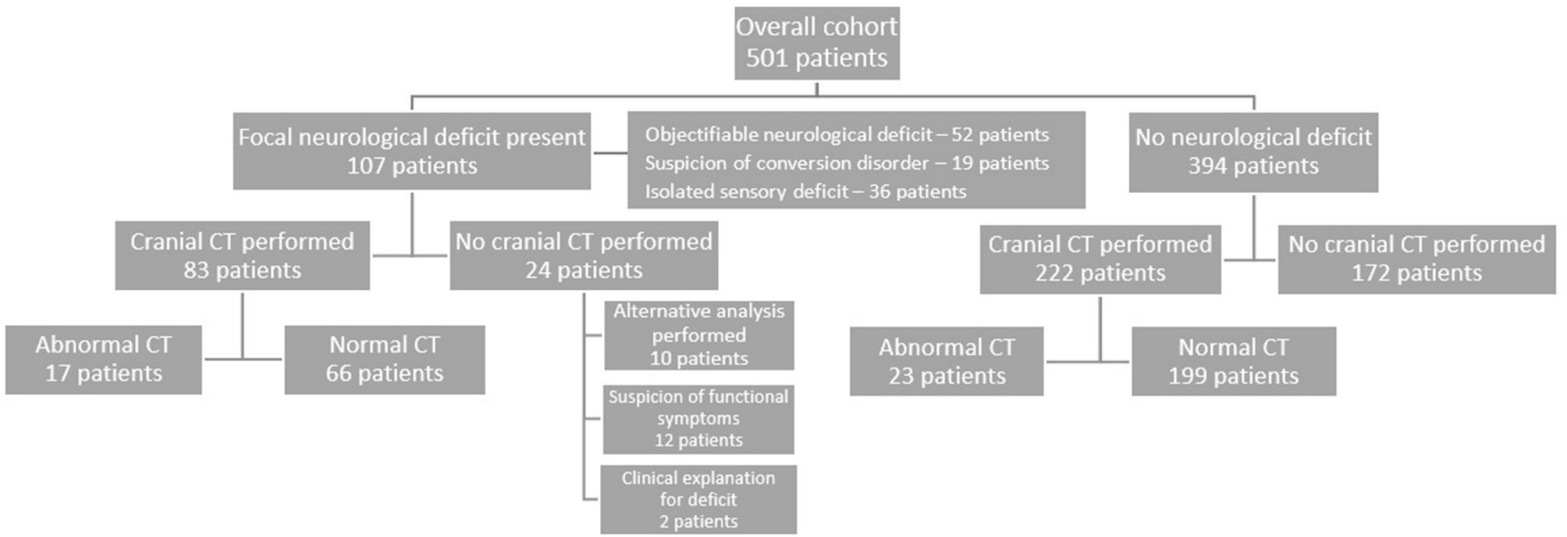
Flowchart of performed cranial CT scans in patients with and without neurological deficit.

Cranial CT scan was performed in 222 out of 394 patients (56%) without neurological deficits at examination; brain abnormalities were found in 23 patients (Figure 1). Out of 107 patients with abnormalities at neurological examination (including suspected functional symptoms), 83 patients (78%) underwent immediate cranial CT imaging of which 17 (20%) demonstrated new intracranial pathology. In 24 patients with abnormal neurological examination who did not receive immediate cranial CT, further analysis was occasionally performed, including lumbar puncture in two patients and delayed cranial MRI in six patients. Sixteen patients who did not require further diagnostic work-up, due to their abnormal neurological examination being characterized as functional neurological symptoms or symptoms, were deemed as of definite peripheral nervous system origin, such a Bell's palsy.

Among patients with neurological deficits without pathology on CT scan were patients with symptoms suspicious of functional neurological symptoms, isolated sensory deficit, and symptoms indicative of vestibular neuritis, post-concussion disorder, and epileptic seizures. In patients with objectifiable neurological deficit, cerebral pathology was present in 16 out of 45 performed scans (36%). In patients with isolated sensory deficit or a suspicion of conversion disorder, brain pathology was found in only one of 40 performed CT scans (3%).

Clinical characteristics significantly associated with abnormal CT results were presentation to the ED within 1 h after headache onset, nausea, clinical history of new aphasia or unilateral paresis, and (objectifiable) focal neurological deficit (Table 1). In the multivariate analysis, four factors were significant predictors of intracranial pathology on CT: age 50 years or older, presentation to the ED within 1 h after headache onset, clinical history of aphasia, and objectifiable focal neurological deficit (Table 3).

**Table 3:** Multivariate analysis of predictors for intracranial pathology on CT imaging.

	**OR**	**95% CI (lower–upper)**	***p*-value**
Age 50 years or older	2.4	1.1–5.0	0.02
Presentation within 1 h after headache onset	5.0	2.0–12.5	<0.001
New aphasia	8.8	3.2–24	<0.001
Objectifiable focal neurological deficit	4.6	2.1–10	<0.001

*OR, odds ratio; CI, confidence interval*.

CTV was performed in 121 patients (24%), detecting cerebral sinus thrombosis in six patients (5%). CTA was performed in 137 patients (27%), which detected abnormalities in 27 patients (including cerebral aneurysm, intracerebral arterial occlusion, signs of reversible cerebral vasoconstriction disorder, arteriovenous malformation, or dissection). In 18 out of 27 patients, non-contrast CT imaging showed signs of subarachnoid hemorrhage, intraparenchymal hemorrhage, or acute cerebral ischemia.

Secondary headache causes were further investigated with cranial MRI in 20 of 265 patients of whom CT scans were normal (8.0%). Intracranial pathology was found in two patients, including hypophysitis and empty sella sign suspect of increased intracranial pressure. Additional lumbar puncture was performed in 35 patients (13%), which detected subarachnoid hemorrhage in two patients and increased intracranial pressure in two other patients.

### Yield of Cranial CT Imaging

For non-contrast-enhanced CT imaging, the number needed to scan (NNS) to detect an intracranial cause of the headache was 7.6. CTA and CTV were associated with an NNS of 5.1 and 20, respectively. In case of objectifiable neurological deficit, the NNS was reduced to 3.

## Discussion

In this study, we present an overview of patients admitted at the ED with non-traumatic headache complaints and the extent to which cranial CT imaging contributes to the diagnosis of a secondary headache disorder. Our results indicate that there is a high tendency to perform cranial imaging in patients presenting with headache at the ED. In our study population, head CT (including CTV and CTA) was obtained in 61% of the cases. The yield of CT imaging was relatively low; on average, 7.6 patients were screened to detect one patient with intracranial pathology. As shown previously, abnormal CT results are rare in headache patients without neurological deficits (10). The extent to which CT scans are performed in headache patients at emergency rooms worldwide varies widely (27–75%) (11–14). Overall, previous research showed a similar low-threshold tendency to perform neuroimaging in the ED setting in both adults and children, with low diagnostic yield (12, 15–18). Our finding of intracranial pathology in 13% of cranial CT scans is in accordance with results presented in previous studies (19–21).

The reason behind the widespread use of neuroimaging in headache patients may be the clinician's fear of misdiagnosing harmful pathology. Another possible contributing factor is patient demand for cranial imaging and the clinician's inclination to meet this demand to improve doctor–patient relations or out of fear of malpractice claims. Moreover, cranial CT imaging has become increasingly available in the ED setting over the last decades. Subsequently, this diagnostic method has acquired the position of a relatively standard part of the ED diagnostic approach in patients with (severe) headache. Excessive implementation of diagnostic procedures may have several negative consequences, as they lead to increased healthcare expenses, radiation risks, longer ED stay of patients, and may unveil intracranial abnormalities with unclear clinical significance.

Previous studies have shown a limited value of clinical characteristics in differentiating primary from secondary headache causes (19, 22). Our study confirms previous observations that focal neurological deficit at examination is the main predictor of intracranial pathology (19, 23, 24). In contrast, patients with isolated sensory deficit or symptoms indicative of conversion disorder were very rarely diagnosed with intracranial pathology. Nevertheless, we emphasize that several secondary causes of headache may present without loss of neurological function. Research showed that 10% of Dutch patients presenting to the general practitioner with acute headache without accompanying symptoms are diagnosed with subarachnoid hemorrhage (25). For these patients with an onset of severe headache presenting within minutes, swift access to cranial CT imaging is critical to avoid missing this life-threatening disease. To select patients at risk of subarachnoid hemorrhage, physicians can rely on clinical risk scores, such as the Ottawa SAH rules (26). Furthermore, we must underline the fact that a considerable number of secondary headache etiologies (infectious disease, increased or decreased intracranial pressure, and subarachnoid hemorrhage >6 h after onset) are not apparent on cranial CT. In case of red flags, such as acute onset of (new) headache, fever, impaired consciousness, or focal neurological deficit, further analysis by lumbar puncture or cranial MRI in the acute setting is warranted. This emphasizes the importance of a thorough neurological assessment of headache patients in the acute setting by an experienced physician.

About half of the patients in our cohort were diagnosed with a primary headache disorder. This finding differs from previous literature, which found a proportion of 58–81% of the cases presenting to the ED for a primary headache disorder (1, 27). In The Netherlands, the general practitioner plays a central role in the triage of patients before presentation to the ED. This may explain the relatively high proportion of patients with a secondary cause for the headache in our cohort.

### Strengths and Limitations

Strengths of our study include the extensive patient population that was analyzed in providing a real-world overview of clinical characteristics, diagnosis, and implementation of diagnostic tests in a Dutch patient cohort presenting with headache as primary complaint at the ED. In addition to other studies, we report the use of cranial MRI and lumbar puncture in this cohort, to elucidate more extensively how secondary headache etiologies were investigated and diagnosed in these patients.

This study has several limitations. First, given the retrospective design, some patients eligible for enrollment may have been missed while constructing the database from the ED registrations, which inherently led to missing data as no standardized case report forms were available. Due to this study design, limited patient follow-up data were available, although in case of outpatient clinic follow-up, the investigator checked for (alterations of) final diagnoses. Second, since this study was performed with single center data and based on local protocols, the findings may not be generalizable to other centers. As population characteristics may differ between medical centers, various prior probabilities may lead to different yields of cranial imaging as found in this study. Third, the prognostic value of the clinical parameters is somewhat limited by the effect of pre-selection since as only 61% of the patients in this cohort underwent CT scanning. Despite these limitations, we attempted to provide a real-world overview of neuroimaging in headache patients at the ED.

### Recommendations for Clinical Practice

A prospective study in patients with non-traumatic headache should be performed to determine specific patient characteristics related with secondary (severe) headache disorders. Previous literature has provided several clinical prediction tools (based on retrospective data) to determine patients' risk of intracranial pathology, which should be externally validated and optimized in different patient populations (19, 24, 26). Evidence-based clinical tools will provide clear diagnostic guidelines for ED clinicians in performing cranial CT in headache patients, which may reduce unnecessary additional imaging and thus healthcare costs.

## Conclusions

Cranial imaging in headache patients at the ED is performed regularly to discriminate between benign and secondary headache disorders. Cranial pathology was detected in our cohort in one out of 7.6 performed scans. Singular clinical characteristics have limited value in detecting cranial pathology. Clinical prediction tools of multiple parameters may guide clinicians in their decision to perform cranial CT in headache patients.

## Data Availability Statement

The raw data supporting the conclusions of this article will be made available by the authors, without undue reservation.

## Ethics Statement

The studies involving human participants were reviewed and approved by Medical Research Ethics Committee (MREC) Leiden The Hague Delft. Written informed consent for participation was not required for this study in accordance with the national legislation and the institutional requirements.

## Author Contributions

All data were retrieved and evaluated by CL. Statistical analysis was performed by CL and ML. All authors contributed to the article and approved the submitted version.

## Conflict of Interest

The authors declare that the research was conducted in the absence of any commercial or financial relationships that could be construed as a potential conflict of interest.

## Abbreviations

CI: confidence interval
CT: computed tomography
CTV: computed tomography venography
CTA: computed tomography angiography
ED: emergency department
MRI: magnetic resonance imaging
NNS: number needed to scan
OR: odds ratio.
